# Molecular profiling of lung cancer specimens and liquid biopsies using MALDI-TOF mass spectrometry

**DOI:** 10.1186/s13000-017-0683-7

**Published:** 2018-01-12

**Authors:** Eleonora Bonaparte, Chiara Pesenti, Laura Fontana, Rossella Falcone, Leda Paganini, Anna Marzorati, Stefano Ferrero, Mario Nosotti, Paolo Mendogni, Claudia Bareggi, Silvia Maria Sirchia, Silvia Tabano, Silvano Bosari, Monica Miozzo

**Affiliations:** 1 Department of Pathophysiology & Transplantation, Università degli Studi di Milano, Via Francesco Sforza, 35 -20122 Milan, Italy; 2 Division of Pathology, Fondazione IRCCS Ca’ Granda Ospedale Maggiore Policlinico, Via Francesco Sforza, 35 –20122 Milan, Italy; 3 Department of Biomedical, Surgical and Dental Sciences, Università degli Studi di Milano, Medical School, Via Francesco Sforza, 35 -20122 Milan, Italy; 4 Thoracic Surgery and Lung Transplantation Unit, Fondazione IRCCS Ca’ Granda Ospedale Maggiore Policlinico, Via Francesco Sforza, 35 -20122 Milan, Italy; 5 Oncology Unit, Fondazione IRCCS Ca’ Granda Ospedale Maggiore Policlinico, Via Francesco Sforza, 35 -20122 Milan, Italy; 6 Medical Genetics, Department of Health Sciences, Università degli Studi di Milano, via Antonio di Rudini, 8 –20142 Milan, Italy

**Keywords:** Molecular diagnostics, MALDI-TOF mass spectrometry, Non-small cell lung cancer, Targeted therapy, Tumor genotyping

## Abstract

**Background:**

Identification of predictive molecular alterations in lung adenocarcinoma is essential for accurate therapeutic decisions. Although several molecular approaches are available, a number of issues, including tumor heterogeneity, frequent material scarcity, and the large number of loci to be investigated, must be taken into account in selecting the most appropriate technique. MALDI-TOF mass spectrometry (MS), which allows multiplexed genotyping, has been adopted in routine diagnostics as a sensitive, reliable, fast, and cost-effective method. Our aim was to test the reliability of this approach in detecting targetable mutations in non-small cell lung cancer (NSCLC). In addition, we also analyzed low-quality samples, such as cytologic specimens, that often, are the unique source of starting material in lung cancer cases, to test the sensitivity of the system.

**Methods:**

We designed a MS–based assay for testing 158 mutations in the *EGFR*, *KRAS*, *BRAF*, *ALK*, *PIK3CA*, *ERBB2*, *DDR2*, *AKT,* and *MEK1* genes and applied it to 92 NSCLC specimens and 13 liquid biopsies from another subset of NSCLC patients. We also tested the sensitivity of the method to distinguish low represented mutations using serial dilutions of mutated DNA.

**Results:**

Our panel is able to detect the most common NSCLC mutations and the frequency of the mutations observed in our cohort was comparable to literature data. The assay identifies mutated alleles at frequencies of 2.5–10%. In addition, we found that the amount of DNA template was irrelevant to efficiently uncover mutated alleles present at high frequency. However, when using less than 10 ng of DNA, the assay can detect mutations present in at least 10% of the alleles.

Finally, using MS and a commercial kit for RT-PCR we tested liquid biopsy from 13 patients with identified mutations in cancers and detected the mutations in 4 (MS) and in 5 samples (RT-PCR).

**Conclusions:**

MS is a powerful method for the routine predictive tests of lung cancer also using low quality and scant tissues. Finally, after appropriate validation and improvement, MS could represent a promising and cost-effective strategy for monitoring the presence and percentage of the mutations also in non-invasive sampling.

**Electronic supplementary material:**

The online version of this article (10.1186/s13000-017-0683-7) contains supplementary material, which is available to authorized users.

## Background

Lung cancer is the leading cause of cancer death worldwide. Non-small cell lung cancers (NSCLCs), primarily adenocarcinoma (ADC) and squamous cell carcinoma (SCC), account for approximately 80% of lung cancer cases [1].

With the introduction of the Epidermal Growth Factor Receptor/Tyrosine Kinase inhibitors (EGFR-TKIs), which target cancer cells harboring activating *EGFR* mutations, the detection of somatic mutations became relevant to treatment choices for lung ADC [2]. Erlotinib, gefitinib, and afatinib are used to target *EGFR*-activating mutations. More recently, the new drug osimertinib was introduced. This molecule can inhibit *EGFR* kinase activity in the presence of the *EGFR* T790 M mutation, which confers resistance to the other inhibitors [3–6]. Yet another drug, crizotinib, inhibits ALK, ROS1, and MET when their kinase activities are aberrantly activated [7–10].

Ongoing clinical trials are investigating emerging agents capable of avoiding acquired tumor resistance to the common TKIs, or of targeting other activated proteins, such as PI3K, AKT1, ERBB2, MEK1, and DDR2 [10, 11].

Mutations in *KRAS* (found in 25–40% of ADC) are a negative prognostic biomarker for NSCLC, since no drugs have been developed to inhibit the mutant protein. Alternative strategies, such as inhibition of MEK, have been suggested as treatment for patients with *KRAS*-mutated cancers [12].

The most frequent activating mutations in lung ADC, other than *KRAS*, involve *EGFR* (15%), whereas *BRAF*, *ERBB2*, and *MEK1* are mutated in less than 2% of cases. *PIK3CA* mutations are present in approximately 1–3% of NSCLCs, and are more common in SCCs (15%). *DDR2* mutations are present in 2% of SCCs. *ALK* and *ROS1* translocations and *MET* amplifications are typical of ADCs, representing 5%, 4%, and 2% of cases, respectively. *AKT1* mutations are found in 1% of lung cancers, more frequently in SCCs [10, 11].

**Fig. 1:**
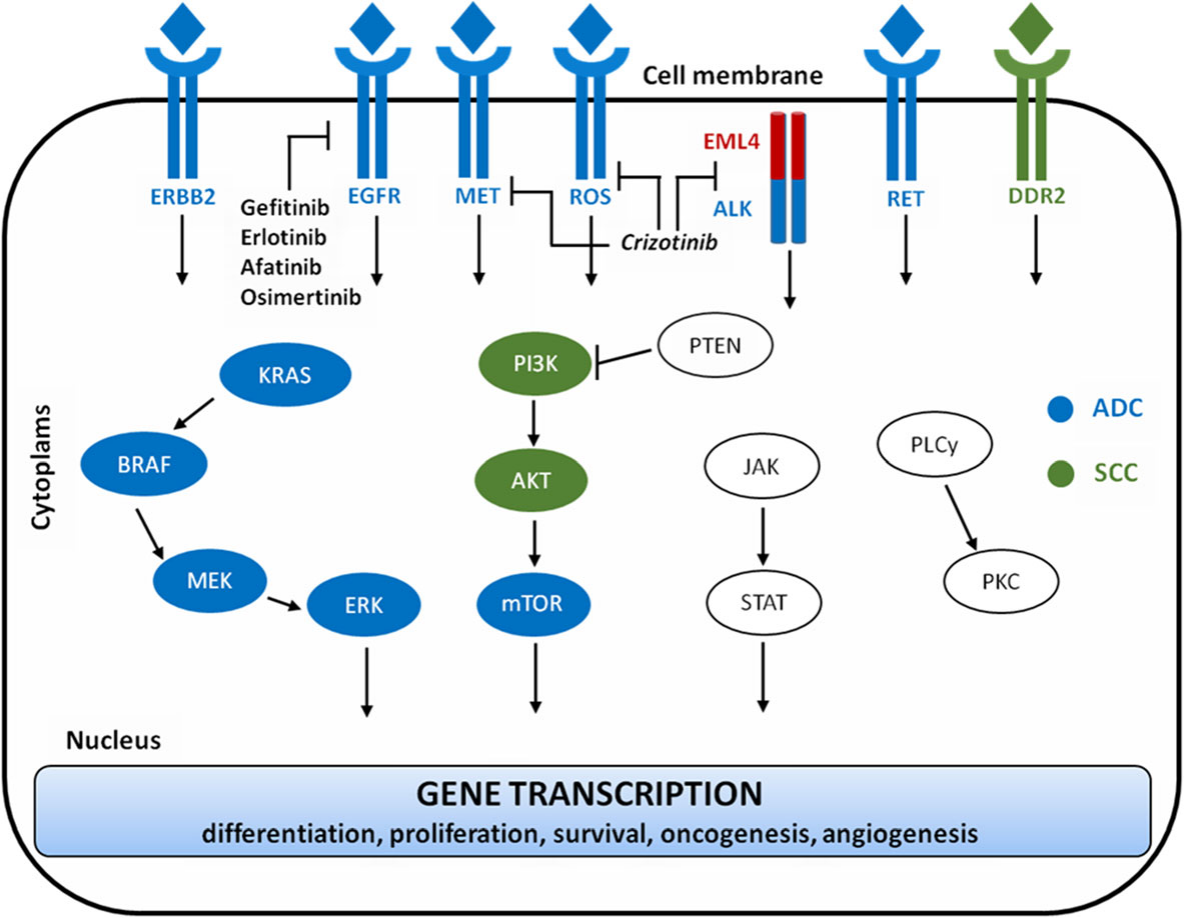
Simplified schema of the most frequently altered signaling pathways in NSCLC. Blue and green ovals indicate the proteins commonly activated in ADC and SSC, respectively. Druggable TKIs and approved targeted agents are specified

A list of druggable molecular markers and pathways in lung cancer is provided in Fig. 1.

Based on the growing knowledge of inhibitors that target abnormally activated kinases and the resultant clinical inclusion of new drugs, the optimal choice of treatment of NSCLC patients relies critically on screening the tumor-related genetic alterations.

Different issues should be taken into account for the molecular characterization of NSCLC. First, NSCLC are heterogeneous and cells harboring a specific mutation may represent a minor clone in a mixture of neoplastic cells, as well as of non-neoplastic, stromal and inflammatory cells [13]. In addition, the availability and/or the quality of the specimens suitable for molecular evaluations could be scare, since formalin-fixed paraffin-embedded (FFPE) samples from small biopsies or cytology specimens could represent the only available material. [14]. Moreover, in recent years, non-invasive approaches (collectively called “liquid biopsy”) have been developed to identify the molecular profile of tumor circulating cells (TCCs) or circulating tumor DNA (ctDNA) [15–18] and therefore very sensitive detection methods are required. For all of these reasons, it is critical to use reliable and sensitive diagnostic methods capable of simultaneously detecting a wide range of mutations also in poor quality samples.

Different molecular approaches, such as Sanger sequencing, real-time PCR, pyrosequencing, MALDI-TOF mass spectrometry (MS) and next-generation sequencing (NGS) are currently available [19–22]. Among them, Sanger sequencing is the less sensitive (at least 20% of mutant alleles); MS is considered a robust approach for the genotyping of known mutations, able to combine the advantages of multiplexing, high sensitivity, and specificity with rapid turnaround, easy sample handling, and cost-effectiveness [19, 20]. Finally, although NGS is a very robust approach with the highest sensitivity [23], it is less affordable than the other approaches and poses several additional challenges, including validation and data handling for diagnostic purposes. Besides, to preserve the cost per test and avoid wasting resources, a consistent number of cases should be simultaneously analyzed.

Using the MS genotyping approach, we tested a cohort of 92 NSCLCs, investigating a wide spectrum of actionable mutations currently targeted by specific therapies or for which clinical trials are ongoing. Our aim was to verify the performance and sensitivity of the method using low levels of tumor DNA. Finally, we evaluated the performance of MS on plasma DNA from 13 lung cancer patients with *EGFR-* or *KRAS*-mutated tumors.

## Methods

### Patients and tumor specimens

The study group included 92 NSCLC cases collected for clinical purposes at the Fondazione IRCCS Ca′ Granda, Ospedale Maggiore Policlinico di Milano (Italy), between September 2011 and December 2013. The molecular evaluation of all cases at the time of diagnosis was carried out by pyrosequencing using Ce-IVD kits (EGFR TKi response (sensitivity), EGFR TKI response (resistance), Anti-EGFR MoAb response KRAS status, Anti-EGFR MoAb response BRAF status - Diatech Pharmacogenetics s.r.l., Jesi, Italy). We included in the study the NSCLC cases for which the biologic material was available. The study was approved by the Institutional Ethic Committee (Fondazione IRCCS Ca′ Granda, Ospedale Maggiore Policlinico di Milano N°526/2015).

For ctDNA analysis, peripheral blood samples were collected from additional 13 lung cancer patients at the time of biopsy/surgical procedures after informed consent. The inclusion criteria were the presence of ADC, the availability of tumor specimens and the positivity for *EGFR* or *KRAS* mutations.

Hematoxylin/eosin-stained sections (H&E) from FFPE tissues were evaluated by a pathologist for routine histopathologic classification and identification of the tumor component. Diagnosis was performed according to the criteria of the 2015 WHO classification for lung tumors [24]_._

Ninety-two NSCLC specimens of primary or metastatic lung tumors, including 28 cytological and 64 histological samples, were classified as follows: 78 ADCs, 11 SCCs, and 3 NSCLCs, not classified more precisely because of the paucity of biological specimens (Table 1). All thirteen cases selected for ctDNA profiling were ADCs.

**Table 1:** Tumor specimens and classification of the 92 NSCLCs cohort

Number of cases	ADC	SSC	NSC
Total	78	11	3
Histological specimens	57	7	0
Cytological specimens	21	4	3
Primary tumors	64	8	3
Metastases	14	3	0

DNAs from NSCLC FFPE samples were obtained using the BiOstic FFPE Tissue DNA Isolation Kit (MO BIO Laboratories Inc., Carlsbad, CA, USA) and quantified using a NanoDrop 1000 UV spectrophotometer, software version 3.7.1 (Thermo Fisher Scientific Inc., Waltham, MA, USA).

ctDNA was extracted with the Helix Circulating Nucleic Acid kit (Diatech Pharmacogenetics, Jesi, Italy) from 3 to 5 ml of plasma obtained from about 10 ml of peripheral blood, collected in EDTA tubes. Following the manufacturer’s instructions, ctDNA was finally eluted in 30 μl and not subsequently quantified.

### MS genotyping assay

A panel of actionable loci was selected based on the Catalogue of Somatic Mutations in Cancer (COSMIC; http://cancer.sanger.ac.uk/cosmic), My Cancer Genome (https://www.mycancergenome.org), and relevant literature [11]. The panel comprised 158 variations affecting commonly mutated genes in NSCLC: *EGFR*, *KRAS*, *BRAF*, *ALK*, *PIK3CA*, *ERBB2*, *DDR2*, *AKT*, and *MEK1*. Reference DNA sequences were retrieved from Ensembl Genome Browser (http://www.ensembl.org/index.html).

Tissue DNAs and ctDNAs were genotyped using the single-base extension technique on a MassARRAY analyzer 4. Amplification and extension primers were designed using the Assay Designer Suite v.1.0 (Agena Bioscience, Hamburg, Germany). Amplification primers were designed with a 10mer tag sequence (lower cases) at 5′-end, to avoid their masses overlapping the range of detection of the MS assay. Primers sequences are available in Additional file 1: Table 1.

The MS panel consists of 48 assays multiplexed in eight wells, testing 158 mutations including base substitutions, deletions, and insertions. A complete description of the mutations is provided in Additional file 2: Table 2. For PCR, SAP (shrimp alkaline phosphatase), and extension reactions, the Complete iPLEX Pro Genotyping Reagent Set (Agena Bioscience) was used. Amplification products were processed using SpectroCHIP II Arrays and Clean Resin Kit and the MassARRAY Nanodispenser (Agena Bioscience). Analyses were performed using the MassARRAY Typer 4.0 software (Agena Bioscience).

**Table 2:** Mutations found in 92 NSCLC FFPE samples

Classification	Total cases	Mutated cases	*EGFR*	*KRAS*	*BRAF*	*ALK*	*PIK3CA*	*ERBB2*	*DDR2*	*AKT*	*MEK1*
ADC	78	45 (57.7%)	12 (15.4%)	29 (37.2%)	1 (1.3%)	–	–	3 (3.8%)	–	2 (2.6%)	–
SCC	11	3 (27.3%)	–	–	–	–	3 (27.3%)	–	–	–	–
NSCLC	3	1 (33.3%)	–	1 (33.3%)	–	–	–	–	–	–	–

Depending on the abundance of each sample, the amount of DNA from FFPE specimens, used as template, was 10–40 ng per well.

### MS assay sensitivity

The analytical sensitivity of the MS assay was determined by verifying the lowest detectable frequency of a mutated allele using commercial reference standards HORIZON (Cambridge, UK) for the following mutations: EGFR G719A, T790 M and L861Q, KRAS Q61L, and BRAF V600E. The standards were heterozygous for the mutations, and thus contained the mutated alleles at 50% frequency. Serial dilutions containing 10%, 5%, and 2.5% of the mutated alleles were obtained by mixing standard samples with wild-type DNA from peripheral blood lymphocytes (PBLs). PCRs were performed using 50 ng of DNA from each dilution.

To determine the minimum amount of DNA needed to detect a mutation present at a low allelic frequency, we used decreasing amounts of DNA (20 ng, 10 ng and 5 ng) from four tumor samples harboring the following mutations at specific percentages: 30% *KRAS* G12C, 20% *KRAS* G12C, 10% *EGFR* L858R, and 9% *PIK3CA* H1047R. These percentages had been assessed previously using 40 ng of template DNA.

### ctDNA genotyping assay

The presence of mutations in ctDNAs was verified using our MS analysis, testing only the assay specific for the mutation previously identified in the tumor sample. To compare MS with another sensitive method, we performed RT-PCR using the commercial IVD-CE kits Easy EGFR and Easy KRAS (Diatech Pharmacogenetics, Jesi, Italy) on Rotor-Gene (Qiagen, Hilden, Germany), following the manufacturer’s instructions. RT-PCR results were analyzed and reported as ΔCt values (Ct sample – Ct wild-type control).

Notably, the amount of the ctDNA requested by RT-PCR and MS was 5 and 2 μl, respectively.

## Results

### Tumor genotyping

We tested 158 actionable mutations comprising base substitutions, insertions, and deletions of the *EGFR*, *KRAS*, *BRAF*, *ALK*, *PIK3CA*, *ERBB2*, *DDR2*, *AKT*, and *MEK1* genes (Additional file 2: Table 2) in 92 NCSLCs and in plasma samples from 13 additional NSCLC patients with known somatic mutations. The mutation profiling of all cases is detailed in Additional file 3: Table 3. The overall data from the 92 NCSLCs revealed that 49 (53.3%) harbored mutations in at least at one gene. In ADCs, we identified mutations in *EGFR* (15.4%), *KRAS* (37.2%), *BRAF* (1.3%), *ERBB2* (3.8%), and *AKT* (2.6%), whereas SCCs exclusively harbored *PIK3CA* mutations (27.3%). Mutations of *ALK*, *DDR2*, and *MEK1* were never detected (Table 2). *EGFR* primarily contained in-frame deletions in exon 19 (6 out of 12 *EGFR* mutated cases). The T790 M and L858R mutations, concomitantly detected in two cases, were the only *EGFR* variants found simultaneously in the same tumor sample (Additional file 3: Table 3).

**Table 3:** MS assay sensitivity tested with reference standard DNA using decreasing percentages of mutated alleles

	Percentages of the mutated allele			
Reference standard	50%	10%	5%	2.5%
EGFR G719A (c.2156 G > C)	D	D	D	D
EGFR L861Q (c.1582 T > A)	D	D	ND	ND
EGFR T790 M (c.2369C > T)	D	D	D	D
KRAS Q61L (c.182 A > T)	D	D	ND	ND
BRAF V600E (c. 1799 T > A)	D	D	ND	ND

D: detected. ND: not detected

*KRAS* was predominantly mutated at codon 12, exon 2 (25 out of 30 *KRAS* mutated cases) (Additional file 3: Table 3).

Only *KRAS* variants were found concomitant with other mutated genes: *AKT* and *ERBB2*, in one case respectively (Additional file 3: Table 3).

Out of the three unclassified NSCLCs, one had *KRAS* G12A mutation (Additional file 3: Table 3), suggesting that it was ADCs since *KRAS* is most frequently mutated in this tumor subtype.

To evaluate the correlation between the mutated alleles percentages detected by MS and the amount of tumor cells at the histopathological evaluation, in two representative mutated ADCs we compared the stained slides with the corresponding MS spectra. As displayed in Additional file 4: Fig. 1, the percentages of mutated alleles are not strictly related to the amount of cancer cells in the samples. Indeed, in the first sample (left), more than 70% of cells are neoplastic, but only a subset of them (23% of the alleles) harbors the EGFR L858R mutation; conversely, in the second ADC (right), the percentage of mutated allele (49%) indicates that about all tumor cells in the sample (tumor content >70%) have the KRAS G12D mutation.

### Genotyping sensitivity

The MS sensitivity was assessed evaluating four common mutations in NSCLC, for which standard commercial references were available. Using 50 ng of HORIZON reference standard DNA (RSs, HORIZON) containing the mutations EGFR G719A, EGFR L861Q, EGFR T790 M, KRAS Q61L, and BRAF V600E in serial dilutions (50%, 10%, 5%, and 2.5%), we found that the sensitivity of MS assays varied depending on the specific mutation tested. For EGFR G719A and T790 M, the sensitivity of the assays was 2.5%, whereas it was 10% for EGFR L861Q, KRAS Q61L, and BRAF V600E (Table 3). These data suggest that MS can detect a mutation with frequency lower than 10%, and that the sensitivity depends on the specific mutation.

**Fig. 2:**
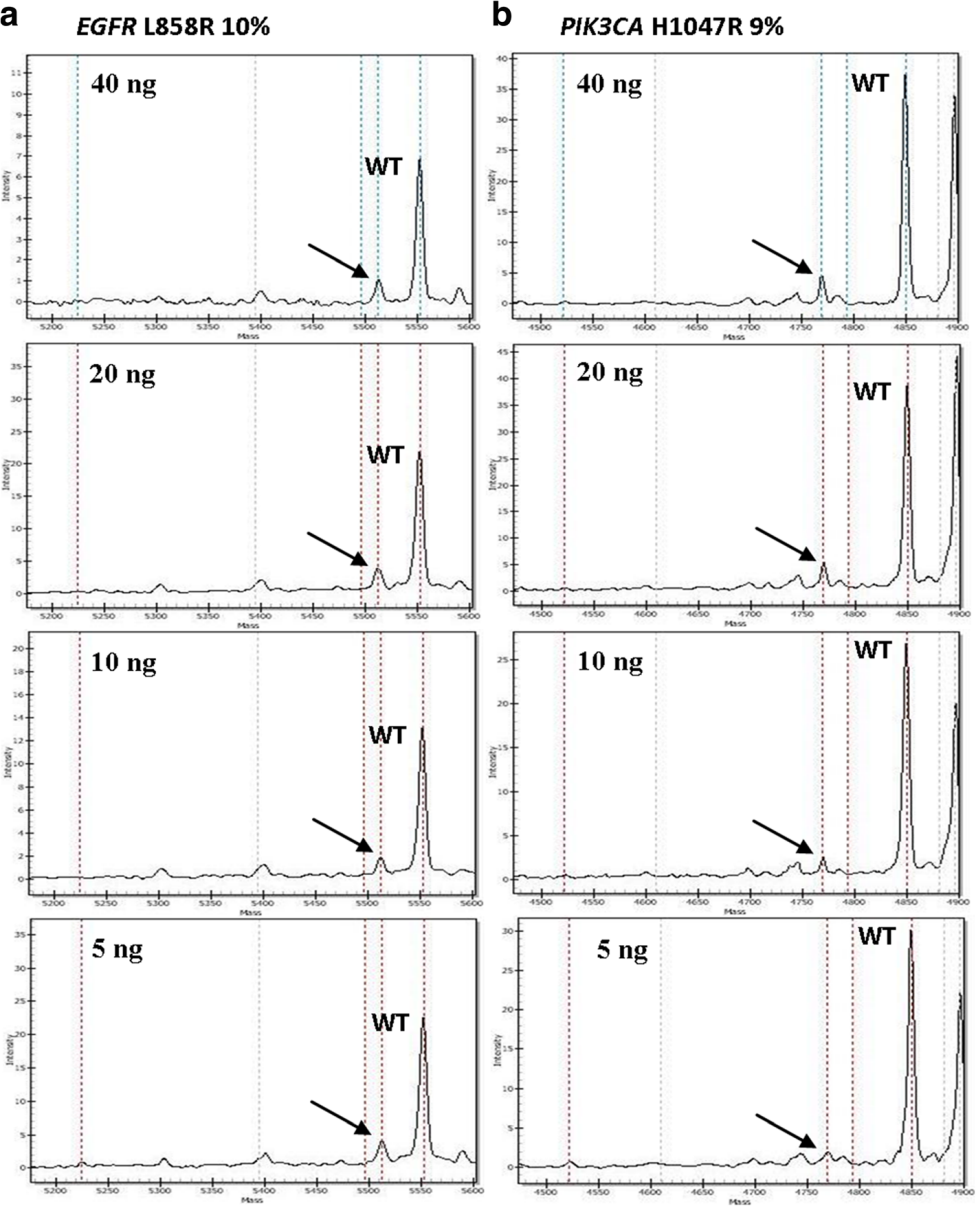
Spectrograms, mass (x-axis) versus intensity (y-axis) from two histological samples (**a**, **b**) containing *EGFR* and *PIK3CA* mutations, respectively, at specific percentages of the mutated alleles (in brackets). Arrows indicate the mass peaks of the mutated alleles, using the specified decreasing amounts of DNA. The mass peaks of the wild-type alleles are also shown (WT). Using 5 ng of tumor DNA, the peak corresponding to the mutations was insufficient for a positive call for *PIK3CA* (**b**), but sufficient for EGFR

In addition, we tested the performance of MS using decreasing amounts of DNA (20 ng, 10 ng, and 5 ng) from four tumor samples harboring mutations at various allelic frequencies (previously identified using 40 ng of DNA). When the frequency of the mutated allele was lower than 10%, the amount of the template DNA influenced the efficiency of detection (Table 4, Fig. 2). Indeed, in cases with *KRAS* G12C and *EGFR* L858R at 30%, 20%, and 10% allelic frequencies, the mutations could be detected irrespective of the amount of DNA (Table 4, Fig. 2). In these cases, the frequency of the mutated alleles remained stable. Conversely, in the H1047R *PIK3CA* sample, with an allelic frequency < 10% (mean value based on triplicate runs: 8% ± 1), the mutated allele could be clearly identified by analyzing 20 ng of DNA, whereas, at lower DNA quantities, the results were uncertain because the signal corresponding to the mass of the mutated analyte was insufficient for a positive call (Table 4, Fig. 2).

**Table 4:** MS assay sensitivity considering various percentages of mutated alleles with serially diluted DNAs from three FFPE sample cases

Template DNA amount (ng)	*KRAS* G12C		*EGFR* L858R	*PIK3CA* H1047R
40	30%	20%	10%	9%
20	D	D	D	D
10	D	D	D	ND
5	D	D	D	ND

D: detected. ND: not detected

These overall data suggest that the sensitivity of MS for detection of point mutation at low levels of mosaicism should be tested for each assay, and that low levels of DNA amounts could affect the recognition of mutations present at low allelic frequencies.

### ctDNA molecular profiling

During the routine molecular evaluation of additional ADCs, we selected thirteen cases harboring mutations of *EGFR* or *KRAS* and analyzed the corresponding ctDNA taken from plasma at the time of biopsy/surgery. Of the thirteen mutations identified in tumor DNA, five (Table 5: cases 1–5) were also detected in plasma DNA by real-time PCR, and four of them (Table 5: cases 1–4) were also detected by MS.

**Table 5:** Mutation detection in ctDNA by real-time PCR and MS approaches

Patients with *EGFR*/*KRAS* mutated tumors	Mutation in FFPE tumors (mutated allele percentage)	Mutation detection in ctDNA by real-time PCR (ΔCt values)	Mutation detection in ctDNA by MS (mutated allele percentage)
1	EGFR L858R (56%)	D (2.55)	EGFR L858R (7%)
2	EGFR E746_A750delELREA (27%)	D (1.44)	EGFR E746_A750delELREA (5%)
3	EGFR E746_A750delELREA (60%)	D (3.66)	EGFR E746_A750delELREA (12%)
4	EGFR E746_A750delELREA (55%)	D (3)	EGFR E746_A750delELREA (19%)
5	EGFR E746_E750delELREA (50%)	D (4.02)	ND
6	EGFR E746_A750delELREA (30%)	ND (12.61)	ND
7	EGFR E746_A750delELREA (30%)	ND (14.03)	ND
8	EGFR L858R (18%)	ND (18.99)	ND
9	KRAS G12 V (50%)	ND (27.23)	ND
10	KRAS G12 V (41%)	ND (18.78)	ND
11	KRAS G12C (17%)	ND (19.63)	ND
12	KRAS G12 V (33%)	ND (27.23)	ND
13	KRAS G12C (20%)	ND (16.95)	ND

D: detected. ND: not detected

In general, the lower detection of mutations in plasma compared to FFPE samples, could be due to the sensitivity limit of the methods and/or to the tumor features (grade, dimension, vascularization, and tissue necrosis), which can affect the amount of tumor DNA released into the bloodstream [25].

The discrepancies between the two approaches were probably not related to the frequencies of mutated alleles in a tumor sample. The percentages of tumor mutated alleles of cases 1–4 were indeed comparable to those of cases 5–13, that were not detected in the plasma (*t-test p* = 0.06) (Table 5), suggesting that the ratio of the mutated alleles in the tumor specimens does not impact their detection by MS assay in plasma samples. The difference in detection rate, between ctDNA and FFPE DNA, could be due to the intrinsic heterogeneity of tumor samples, which could in turn be related to the different amounts of ctDNA released [26].

In plasma samples the range of the mutated alleles frequency detected by MS was 5–19%. RT-PCR does not allow the alleles percentages, a data that could have a clinical significance in monitoring patients.

## Discussion

A large number of proteins activated in cancer and involved in the intracellular signaling pathways have been investigated as approved or potential targets for biological inhibitors. These discoveries led to a significant increase in molecular testing on tumor samples. Consequently, it becomes crucial to select a robust diagnostic method capable of identifying a wide spectrum of mutations in low-quality samples and in a cost-effective manner. We investigated whether MS would be reliable for this purpose, considering several challenges of tumor genotyping: the frequent paucity of available biological material, the low yield of DNA from FFPE specimens or liquid biopsy, and tumor heterogeneity, which is associated with variable frequencies of somatic mosaicism.

We found that mutations at 10% frequency could always be detected using our multiplexed genotyping panel, which covers 158 mutations in six genes. However, we confirmed that the sensitivity of MS depended on the specific assay, and genetic alterations could also be detected when the mutated allele frequency is very low, e.g., 5% or 2.5% [19, 20]. On this basis, assuming that mutations are heterozygous, a 10% mutated allele frequency corresponds to 20% of cells carrying the mutation [27]. In turn, this implies that a minimum of 20% of tumor cells in a tissue specimen should be required to detect mutations at 10%. However, considering the tumor heterogeneity, it is also possible that a target mutation could be present only in a subset of cancer cells, and thus identifiable by MS, depending on MS performance for each mutation and the DNA quality.

Another crucial issue is the minimum amount of DNA needed for analysis. Frequently, the available amount of DNA is limited by the small sizes of biopsies or cytology specimens. We observed a relationship between the frequency of a mutated allele in the sample and the amount of DNA required for detection. This is partially in contrast to the findings of Magliacane et al. (2015), who reported that mutations could be detected even using very small amounts of DNA (1 ng per reaction) [28]. In particular, we observed that, in the presence of highly represented mutations (about 20%), only a very small amount of DNA (about 5 ng) was sufficient for the detection. By contrast, the quantity of DNA is crucial when identifying mutations with a frequency lower than 10%. Mutations represented at 10% and 30% were detected in all cases, but, when the mutated allele frequency was lower than 10%, the amount of template DNA influenced the performance of the MS analysis.

Taken together, these results showed that, to define the robustness of molecular profiling using MS, it is necessary to set the sensitivity of the method for each mutation to be investigated.

The validity of the mutation detection by MS was confirmed by comparison with the results reported in the literature: *KRAS* is the most frequently mutated gene in ADCs (37.2%), followed by *EGFR* (14.4%), whereas *PIK3CA* is frequently mutated in SCCs (27.3%) [10].

Three NSCLC cases were not further classified because of the paucity of specimens. Our molecular results identified the *KRAS* G12A mutation in one of them, suggesting that it could be classified as ADCs. This case confirms the importance of molecular profiling evaluation in lung cancer to complete the histopathological diagnosis, as already indicated for brain tumors [29].

In summary, MS allows the genotyping of several samples simultaneously by screening many known mutations in a single and cost effective test. It has high sensitivity, an important feature when a minority of mutant alleles must be distinguished from abundant wild-type alleles, and also allows mutations to be detected from a small amount of low-quality DNA, such as that typically obtained from poor-quality tissue specimens. Therefore, our experience emphasizes that it may not be appropriate to decline to perform molecular diagnosis on tissue specimens with a tumor component lower than 20%, or on poor biological materials.

Regarding the ctDNA evaluation, we observed concordant mutation detection between tumor tissue and plasma in four out of thirteen cases using MS, whereas, when using real-time PCR, we detected the tumor mutation in an additional case. The low sensitivity of both methods could be due to the low level of the mutated allele in the bloodstream or to tumor heterogeneity, although the small number of analyzed cases prevents us from drawing definitive conclusions. MS seems to be quite less sensitive than real-time PCR; however, MS has the advantage to screen panels of variations simultaneously. Our preliminary data suggest that, although in some cases analysis of ctDNA alone can be insufficient, MS has the potential to analyze liquid biopsy and monitor patients during treatment. Moreover, MS allows the estimation of alleles percentages, a data that could have a clinical significance in monitoring patients.

## Conclusions

Our paper provides important advises for the use of MS in predictive analyses.

By analyzing 158 sequence alterations of the most frequently NSCLCs mutated genes in 92 tumor specimens, we confirmed that the method is able to detect mutated alleles present at 2,5% in the tumor specimens, and that the sensitivity can vary, depending on the mutation analyzed [19].

Interestingly, we noticed that the amount of DNA could affect the analysis. Specifically, when the mutation is present in more than 10% of alleles it is detectable even using low DNA amount (5 ng), but when the mutated alleles are less than 10%, the mutation detection can be compromised when using as low as 10 ng of DNA.

Finally, a proof-of concept investigation on liquid biopsy testing suggests that MS can be a reliable approach for this purpose even though it needs to be improved.

MS is a powerful and high throughput method for detecting known mutations, and allows to genotype scarce component of tumor cells in the tissue specimen, this has an important impact on patient clinical management.

## Additional files

Additional file 1: Table S1. Amplification and extension primer sequences for MS panel. (DOCX 16 kb)

Additional file 2: Table S2. List of alterations (base substitutions, deletions, and insertions) included in the MS panel. On the left, mutations not distinguishable from one another are indicated by the same number. (DOCX 30 kb)

Additional file 3: Table S3. Molecular profile of NSCLC mutated cases. For each detected mutation, the number of positive cases and the diagnosis are reported. (DOCX 14 kb)

Additional file 4: Figure 1. H&E staining of two ADCs cases and the corresponding mutation MS spectra. **A** H&E staining of two ADCs cases. 5X zoom of the rectangular area is shown. The percentage of cancer cells is higher than 70% in both samples. **B** MS spectra of the two ADCs cases harboring EGFR L858R and KRAS G12D mutations, respectively. The mutated alleles are pointed out by black arrows and the corresponding percentages are reported in each spectrum. (TIFF 26330 kb)

## Abbreviations

ADC: adenocarcinoma
AKT: AKT serine/threonine kinase 1
ALK: anaplastic lymphoma kinase
BRAF: B-Raf proto-oncogene, serine/threonine kinase;
COSMIC: catalogue of somatic mutations in cancer
ctDNA: circulating tumor DNA
DDR2: discoidin domain receptor tyrosine kinase 2
EGFR: epidermal growth factor receptor
EGFR-TKIs: epidermal growth factor receptor/tyrosine kinase inhibitors
ERBB2: human epidermal growth factor receptor 2
FFPE: formalin-fixed paraffin-embedded
H&E: (Hematoxylin & Eosin staining).
KRAS: Kirsten rat sarcoma viral oncogene homolog
MEK1: mitogen-activated protein kinase kinase 1
MET: MET proto-oncogene, receptor tyrosine kinase
MS: MALDI-TOF mass spectrometry
NGS: next-generation sequencing
NSCLC: non-small cell lung cancers
PI3K: phosphoinositide 3-kinase
PIK3CA: phosphatidylinositol-4,5-bisphosphate 3-kinase catalytic subunit alpha
ROS1: ROS proto-oncogene 1, receptor tyrosine kinase
SAP: shrimp alkaline phosphatase
SCC: squamous cell carcinoma
TCCs: tumor circulating cells
