# Supplementary material for: Molecular profiling of lung cancer specimens and liquid biopsies using MALDI-TOF mass spectrometry
Source: Diagn Pathol. 2018 Jan 12;13:4. doi: 10.1186/s13000-017-0683-7 (PMC6389067; doi:10.1186/s13000-017-0683-7)
Supplement: Supplementary file 1 — Amplification and extension primer sequences for MS panel. (DOCX 16 kb) [file 13000_2017_683_MOESM1_ESM.docx]

Supplementary Table 1. Amplification and extension primer sequences for MS panel.

| **Amplification primers** | **Sequence** |
| --- | --- |
| EGFR_cod719_F | 5'-acgttggatgTCTCTTGAGGATCTTGAAGG-3' |
| EGFR_cod719_R | 5'-acgttggatgTTACCTTATACACCGTGCCG-3' |
| EGFR_DEL19_F | 5'-acgttggatgTCTCTCTGTCATAGGGACTC-3' |
| EGFR_DEL19_R | 5'-acgttggatgTGAGGTTCAGAGCCATGGAC-3' |
| EGFR_cod768_F | 5'-acgttggatgTGCGAAGCCACACTGACGT-3' |
| EGFR_cod768_R | 5'-acgttggatgGATGAGCTGCACGGTGGA-3' |
| EGFR_cod790_F | 5'-acgttggatgGCATCTGCCTCACCTCCACC-3' |
| EGFR_cod790_R | 5'-acgttggatgAGCCAATATTGTCTTTGTGT-3' |
| EGFR_2310_2311_F | 5'-acgttggatgTCCAGGAAGCCTACGTGATG-3' |
| EGFR_2310_2311_R | 5'-acgttggatgATGAGCTGCGTGATGAGCTG-3' |
| EGFR_cod858-861_F | 5'-acgttggatgAAACACCGCAGCATGTCAAG-3' |
| EGFR_cod858-861_R | 5'-acgttggatgCCTCCTTCTGCATGGTATTC-3' |
| KRAS_cod12_13_F | 5'-acgttggatgAGGCCTGCTGAAAATGACTG-3' |
| KRAS_cod12_13_R | 5'-acgttggatgCTGTATCGTCAAGGCACTCT-3' |
| KRAS_cod61_F | 5'-acgttggatgTGGAGAAACCTGTCTCTTGG-3' |
| KRAS_cod61_R | 5'-acgttggatgCATGTACTGGTCCCTCATTG-3' |
| KRAS_146_F | 5'-acgttggatgCAGGCTCAGGACTTAGCAAG-3' |
| KRAS_146_R | 5'-acgttggatgTTCAGTGTTACTTACCTGTC-3' |
| BRAF_cod600_F | 5'-acgttggatgTCTTCATGAAGACCTCACAG-3' |
| BRAF_cod600_R | 5'-acgttggatgTGGATCCAGACAACTGTTC-3' |
| ALK_cod-1196-1202-1206_F | 5'-acgttggatgACATTGTTCGCTGCATTGGG-3' |
| ALK_cod-1196-1202-1206_R | 5'-acgttggatgAGACTGGTTCTCACTCACCG-3' |
| ALK_cod1269_F | 5'-acgttggatgAGAAACTGCCTCTTGACCTG-3' |
| ALK_cod1269_R | 5'-acgttggatgACTCACCTGTAGATGTCTCG-3' |
| AKT1_cod17_F | 5'-acgttggatgTCTGACGGGTAGAGTGTGC-3' |
| AKT1_cod17_R | 5'-acgttggatgTTCTTGAGGAGGAAGTAGCG-3' |
| DDR2_cod768_F | 5'-acgttggatgTTAACAGGGTGTTGTTGTGC-3' |
| DDR2_cod768_R | 5'-acgttggatgCACAAAGTAACCCCAAAGGC-3' |
| ERBB2_2264_2305_F | 5'-acgttggatgTGAAAATTCCAGTGGCCATC-3' |
| ERBB2_2264_2305_R | 5'-acgttggatgTCCTTCCTGTCCTCCTAGCA-3' |
| ERBB2_2324_2325_F | 5'-acgttggatgTCTCAGCGTACCCTTGTCC-3' |
| ERBB2_2324_2325_R | 5'-acgttggatgAGAAGGCGGGAGACATATGG-3' |
| ERBB2_2325_2326_F | 5'-acgttggatgCCTTGTCCCCAGGAAGCATA-3' |
| ERBB2_2325_2326_R | 5'-acgttggatgAGAAGGCGGGAGACATATGG-3' |
| ERBB2_2326_2327_2331_2332_F | 5'-acgttggatgTTGTCCCCAGGAAGCATACG-3' |
| ERBB2_2326_2331_2332_R | 5'-acgttggatgAGAAGGCGGGAGACATATGG-3' |
| ERBB2_2339_2340_F | 5'-acgttggatgCATACGTGATGGCTGGTGTG-3' |
| ERBB2_2339_2340_R | 5'-acgttggatgCATAGGGCATAAGCTGTGTC-3' |
| MEK1_cod-56-57-67_F | 5'-acgttggatgAAAGCGCCTTGAGGCCTTTC-3' |
| PIK3CA_542-545_F | 5'-acgttggatgGCAATTTCTACACGAGATCC-3' |
| PIK3CA_542-545_R | 5'-acgttggatgTAGCACTTACCTGTGACTCC-3' |
| PIK3CA_1047_F | 5'-acgttggatgAACTGAGCAAGAGGCTTTGG-3' |
| PIK3CA_1047_R | 5'-acgttggatgTCCATTTTTGTTGTCCAGCC-3' |
| **Extension primers** | **Sequence** |
| EGFR_cod719_ca2r_E | 5'-ACCGTGCCGAACGCACCGGAGC-3' |
| EGFR_cod719_car_II_E | 5'-ACCGTGCCGAACGCACCGGAG-3' |
| EGFR_DEL19_FW_2235_E | 5'-AAGTTAAAATTCCCGTCGCTATCAA-3' |
| EGFR_DEL19_FW_2236_E | 5'-TAAAATTCCCGTCGCTATCAAG-3' |
| EGFR_DEL19_FW_2239_E | 5'-TCCCGTCGCTATCAAGGAA-3' |
| EGFR_DEL19_FW_2240_E | 5'-ATTCCCGTCGCTATCAAGGAAT-3' |
| EGFR_DEL19_REV_2249_E | 5'-CTTGTTGGCTTTCGGAGATGTT-3' |
| EGFR_DEL19_REV_2250_E | 5'-CCTTGTTGGCTTTCGGAGATGT-3' |
| EGFR_DEL19_REV_2251_E | 5'-TCCTTGTTGGCTTTCGGAGATG-3' |
| EGFR_DEL19_REV_2252_E | 5'-TTCCTTGTTGGCTTTCGGAGAT-3' |
| EGFR_DEL19_REV_2255_E | 5'-GATTTCCTTGTTGGCTTTCGGA-3' |
| EGFR_DEL19_REV_2257_E | 5'-ATCGAGGATTTCCTTGTTGGCTTTCG-3' |
| EGFR_cod768_E | 5'-AAGCCTACGTGATGGCCA-3' |
| EGFR_cod790_E | 5'-CGAAGGGCATGAGCTGC-3' |
| EGFR_2310_2311_E | 5'-GATGGCCAGCGTGGAC-3' |
| EGFR_cod858_E | 5'-GATCACAGATTTTGGGC-3' |
| EGFR_cod861_E | 5'-CTTTCTCTTCCGCACCCAGC-3' |
| KRAS_cod12_caf | 5'-CTTGTGGTAGTTGGAGCT-3' |
| KRAS_cod12_car_II_E | 5'-ACTCTTGCCTACGCCA-3' |
| KRAS_cod13_ca2r_E | 5'-AAGGCACTCTTGCCTACGC-3' |
| KRAS_cod13_car_II_E | 5'-CAAGGCACTCTTGCCTACG-3' |
| KRAS_cod61_caf_E | 5'-CTCGACACAGCAGGT-3' |
| KRAS_cod61_ca2f_E | 5'-TCTCGACACAGCAGGTC-3' |
| KRAS_cod61_car_E | 5'-CATTGCACTGTACTCCTC-3' |
| KRAS_146_caf_E | 5'-GGAATTCCTTTTATTGAAACATCA-3' |
| KRAS_146_car_II_E | 5'-CTTACCTGTCTTGTCTTT-3' |
| BRAF_V600_caf_E | 5'-GTGATTTTGGTCTAGCTACA-3' |
| BRAF_V600_car_E | 5'-CCACTCCATCGAGATTTC-3' |
| ALK_cod1196_E | 5'-CCCCGCCATGAGCTCCA-3' |
| ALK_cod1202_E | 5'-GGAGCTCATGGCGGGG-3' |
| ALK_cod1206_E | 5'-GGTCTCTCGGAGGAAG-3' |
| ALK_cod1269_E | 5'-AAGAGTGGCCAAGATTG-3' |
| AKT1_cod17_E | 5'-CGCACGTCTGTAGGG-3' |
| DDR2_cod768_E | 5'-CAAGTTCACTACAGCAAG-3' |
| ERBB2_2264_E | 5'-TGGCCATCAAAGTGT-3' |
| ERBB2_2305_E | 5'-AAGCCAACAAAGAAATCTTA-3' |
| ERBB2_2324_2325_E | 5'-AGCATACGTGATGGC-3' |
| ERBB2_2325_2326_E | 5'-GCATACGTGATGGCT-3' |
| ERBB2_2326_E | 5'-TATGGGGAGCCCACAC-3' |
| ERBB2_2326_2327_E | 5'-GAAGCATACGTGATGGCTG-3' |
| ERBB2_2331_2332_E | 5'-TACGTGATGGCTGGTGTG-3' |
| ERBB2_2339_2340_E | 5'-ATGGCTGGTGTGGGCTCCCC-3' |
| MEK1_cod56_E | 5'-CTTGAGGCCTTTCTTACCC-3' |
| MEK1_cod57_E | 5'-GAGGCCTTTCTTACCCAGAA-3' |
| MEK1_cod67_E | 5'-GGAGAACTGAAGGATGAC-3' |
| PIK3CA_542_E | 5'-TACACGAGATCCTCTCTCT-3' |
| PIK3CA_545_E | 5'-TAGAAAATCTTTCTCCTGCT-3' |
| PIK3CA_1047_E | 5'-GTCCAGCCACCATGA-3' |
