# Supplementary material for: Molecular profiling of lung cancer specimens and liquid biopsies using MALDI-TOF mass spectrometry
Source: Diagn Pathol. 2018 Jan 12;13:4. doi: 10.1186/s13000-017-0683-7 (PMC6389067; doi:10.1186/s13000-017-0683-7)
Supplement: Supplementary file 2 — List of alterations (base substitutions, deletions, and insertions) included in the MS panel. On the left, mutations not distinguishable from one another are indicated by the same number. (DOCX 30 kb) [file 13000_2017_683_MOESM2_ESM.docx]

Supplementary Table 2. List of alterations (base substitutions, deletions, and insertions) included in the MS panel. On the left, mutations not distinguishable from one another are indicated by the same number.

| Gene | Exon | CDS Mutation | AA Mutation | Mutations not distinguishable |
| --- | --- | --- | --- | --- |
| *EGFR* | 18 | c.2155G>A | p.G719S |  |
| *EGFR* | 18 | c.2155G>T | p.G719C |  |
| *EGFR* | 18 | c.2156G>A | p.G719D |  |
| *EGFR* | 18 | c.2156G>C | p.G719A |  |
| *EGFR* | 19 | c.2230_2249>GTCAA | p.I744_A750>VK |  |
| *EGFR* | 19 | c.2232_2249del18 | p.K745_A750delKELREA |  |
| *EGFR* | 19 | c.2234_2235ins18 | p.K745_E746insIPVAIK | 1 |
| *EGFR* | 19 | c.2234_2235ins18 | p.K745_E746insTPVAIK | 1 |
| *EGFR* | 19 | c.2235_2246del12 | p.E746_E749delELRE | 1 |
| *EGFR* | 19 | c.2235_2248>AATTC | p.E746_T750>IP | 1 |
| *EGFR* | 19 | c.2235_2249del15 | p.E746_A750delELREA |  |
| *EGFR* | 19 | c.2235_2251>AATTC | p.E746_T751>IP |  |
| *EGFR* | 19 | c.2235_2251>AG | p.E746_T751>A |  |
| *EGFR* | 19 | c.2235_2252>AAT | p.E746_S751>I |  |
| *EGFR* | 19 | c.2235_2252del18 | p.E746_T751delELREAT |  |
| *EGFR* | 19 | c.2235_2255>AAT | p.E746_S752>I |  |
| *EGFR* | 19 | c.2235_2255>GGT | p.E746_S752>V | 2 |
| *EGFR* | 19 | c.2236G>A | p.E746K | 3 |
| *EGFR* | 19 | c.2236_2248>AGAC | p.E746_A750>RP | 3 |
| *EGFR* | 19 | c.2236_2248>CAAC | p.E746_A750>QP | 4 |
| *EGFR* | 19 | c.2236_2250del15 | p.E746_A750delELREA |  |
| *EGFR* | 19 | c.2236_2251>T | p.E746_T751>S |  |
| *EGFR* | 19 | c.2236_2252>AT | p.E746_S751>I |  |
| *EGFR* | 19 | c.2236_2253>ATTCCT | p.E746_T751>IP | 3 |
| *EGFR* | 19 | c.2236_2253>CAA | p.E746_T751>Q |  |
| *EGFR* | 19 | c.2236_2253del18 | p.E746_T751delELREAT |  |
| *EGFR* | 19 | c.2236_2255>AT | p.E746_S752>I |  |
| *EGFR* | 19 | c.2236_2256del21 | p.E746_S752delELREATS | 4 |
| *EGFR* | 19 | c.2237_2250>TCCCT | p.E746_T750>VP |  |
| *EGFR* | 19 | c.2237_2251 del15 | p.E746_T751>A | 5 |
| *EGFR* | 19 | c.2237_2251>TTC | p.E746_T751>VP |  |
| *EGFR* | 19 | c.2237_2251>TGG | p.E746_T751>VA | 5 |
| *EGFR* | 19 | c.2237_2252>T | p.E746_T751>V | 6 |
| *EGFR* | 19 | c.2237_2253>TA | p.E746_T751>V | 6 |
| *EGFR* | 19 | c.2237_2255>T | p.E746_S752>V | 2 |
| *EGFR* | 19 | c.2237_2257>TCT | p.E746_P753>VS | 7 |
| *EGFR* | 19 | c.2238_2252>GCA | p.L747_T751>Q |  |
| *EGFR* | 19 | c.2238_2252del15 | p.L747_T751delLREAT | 8 |
| *EGFR* | 19 | c.2238_2255del18 | p.E746_S752>D | 9 |
| *EGFR* | 19 | c.2238_2255>GCAACA | p.L747_S752>QH | 9 |
| *EGFR* | 19 | c.2239_2240TT>CC | p.L747P | 10 |
| *EGFR* | 19 | c.2239_2247del9 | p.L747_E749delLRE | 11 |
| *EGFR* | 19 | c.2239_2248>C | p.L747_A750>P | 10 |
| *EGFR* | 19 | c.2239_2250>CCA | p.L747_A750>P | 10 |
| *EGFR* | 19 | c.2239_2251>C | p.L747_S751>P |  |
| *EGFR* | 19 | c.2239_2252>CA | p.L747_T751>Q | 12 |
| *EGFR* | 19 | c.2239_2253>CAA | p.L747_T751>Q | 12 |
| *EGFR* | 19 | c.2239_2253del15 | p.L747_T751delLREAT | 8 |
| *EGFR* | 19 | c.2239_2253>CCAACG | p.L747_T751>PT | 10 |
| *EGFR* | 19 | c.2239_2253>GCT | p.L747_T751>A | 11 |
| *EGFR* | 19 | c.2239_2256del18 | p.L747_S752del | 10 |
| *EGFR* | 19 | c.2239_2256>CAACAT | p.L747_S752>QH |  |
| *EGFR* | 19 | c.2239_2256>CAA | p.L747_S752>Q | 10 |
| *EGFR* | 19 | c.2239_2257>T | p.L747_P753>S | 7 |
| *EGFR* | 19 | c.2239_2258>CA | p.L747_P753>Q | 10 |
| *EGFR* | 19 | c.2239_2262del24 | p.L747_K754del | 11 |
| *EGFR* | 19 | c.2239_2264>GCCAA | p.L747_A755>AN | 11 |
| *EGFR* | 19 | c.2240T>C | p.L747S | 13 |
| *EGFR* | 19 | c.2240T>A | p.L747T |  |
| *EGFR* | 19 | c.2240-2251 del12 | p.L747_T751>S |  |
| *EGFR* | 19 | c.2240_2254del15 | p.L747_T751delLREAT | 8 |
| *EGFR* | 19 | c.2240-2257 del18 | p.L747_P753>S |  |
| *EGFR* | 19 | c.2240_2261>CGAC | p.L747_K754>ST | 13 |
| *EGFR* | 19 | c.2240_2264>CGAAAGG | p.L747_A755>SKG | 13 |
| *EGFR* | 19 | c.2252C>T | p.T751I | 6 |
| *EGFR* | 19 | c.2255A>C | p.S752Y | 9 |
| *EGFR* | 19 | c.2257C>T | p.P753S | 7 |
| *EGFR* | 20 | c.2302_2303ins9 | p.A767_S768insTLA |  |
| *EGFR* | 20 | c.2303G>A | p.S768N |  |
| *EGFR* | 20 | c.2303G>T | p.S768I | 14 |
| *EGFR* | 20 | c.2310_2311insTAC | p.D770_N771insY |  |
| *EGFR* | 20 | c.2310_2311insGGT | p.D770_N771insG | 15 |
| *EGFR* | 20 | c.2310_2311insGGC | p.D770_N771insG | 15 |
| *EGFR* | 20 | c.2310_2311insGCACCGTGG | D770_N771insAPW | 15 |
| *EGFR* | 20 | c.2310_2311insGGCACA | D770_N771insGT | 15 |
| *EGFR* | 20 | c.2310_2311insGGGGAC | D770_N771insGD | 15 |
| *EGFR* | 20 | c.2310_2311insGGGTTA | D770_N771insGL | 15 |
| *EGFR* | 20 | c.2310_2311insGGGTTT | D770_N771insGF | 15 |
| *EGFR* | 20 | c.2369C>T | p.T790M |  |
| *EGFR* | 21 | c.2573T>G | p.L858R | 16 |
| *EGFR* | 21 | c.2573_2574TG>GT | p.L858R | 16 |
| *EGFR* | 21 | c.2573_2574TG>GA | p.L858R | 16 |
| *EGFR* | 21 | c.2573T>A | p.L858Q |  |
| *EGFR* | 21 | c.2582T>A | p.L861Q |  |
| *EGFR* | 21 | c.2582T>G | p.L861R |  |
| *KRAS* | 2 | c.34G>A | p.G12S |  |
| *KRAS* | 2 | c.34G>C | p.G12R |  |
| *KRAS* | 2 | c.34G>T | p.G12C |  |
| *KRAS* | 2 | c.34_35GG>AT | p.G12I |  |
| *KRAS* | 2 | c.34_35GG>TT | p.G12F |  |
| *KRAS* | 2 | c.34_35GG>TA | p.G12Y |  |
| *KRAS* | 2 | c.34_35GG>AA | p.G12N |  |
| *KRAS* | 2 | c.35G>A | p.G12D | 17 |
| *KRAS* | 2 | c.35_36GT>AA | p.G12E | 17 |
| *KRAS* | 2 | c.35_36GT>AC | p.G12D | 17 |
| *KRAS* | 2 | c.35G>T | p.G12V | 18 |
| *KRAS* | 2 | c.35_36GT>TC | p.G12V | 18 |
| *KRAS* | 2 | c.35G>C | p.G12A |  |
| *KRAS* | 2 | c.37G>T | p.G13C |  |
| *KRAS* | 2 | c.37G>A | p.G13S |  |
| *KRAS* | 2 | c.37G>C | p.G13R |  |
| *KRAS* | 2 | c.38G>A | p.G13D |  |
| *KRAS* | 2 | c.38G>C | p.G13A |  |
| *KRAS* | 2 | c.38G>T | p.G13V |  |
| *KRAS* | 3 | c.181C>A | p.Q61K |  |
| *KRAS* | 3 | c.181C>G | p.Q61E |  |
| *KRAS* | 3 | c.182A>C | p.Q61P |  |
| *KRAS* | 3 | c.182A>T | p.Q61L |  |
| *KRAS* | 3 | c.182A>G | p.Q61R |  |
| *KRAS* | 3 | c.183A>C | p.Q61H |  |
| *KRAS* | 3 | c.183A>T | p.Q61H |  |
| *KRAS* | 4 | c.436G>A | p.A146T |  |
| *KRAS* | 4 | c.436G>C | p.A146P |  |
| *KRAS* | 4 | c.436G>T | p.A146S |  |
| *KRAS* | 4 | c.437C>G | p.A146G |  |
| *KRAS* | 4 | c.437C>A | p.A146E |  |
| *KRAS* | 4 | c.437C>T | p.A146V |  |
| *PIK3CA* | 10 | c.1624G>A | p.E542K |  |
| *PIK3CA* | 10 | c.1633G>A | p.E545K |  |
| *PIK3CA* | 10 | c.1633G>C | p.E545Q |  |
| *PIK3CA* | 21 | c.3140A>T | p.H1047L |  |
| *PIK3CA* | 21 | c.3140A>G | p.H1047R |  |
| *BRAF* | 15 | c.1798G>A | p.V600M | 19 |
| *BRAF* | 15 | c.1797_1799AGT>GAG | p.V600R | 19 |
| *BRAF* | 15 | c.1798G>T | p.V600L |  |
| *BRAF* | 15 | c.1798_1799GT>AG | p.V600R |  |
| *BRAF* | 15 | c.1798-1799GT>CG | p.V600R |  |
| *BRAF* | 15 | c.1798-1799GT>AA | p.V600K |  |
| *BRAF* | 15 | c.1799T>A | p.V600E | 20 |
| *BRAF* | 15 | c.1799-1800 TG>AA | p.V600E2 | 20 |
| *BRAF* | 15 | c.1799-1800 TG>AT | p.V600D | 20 |
| *BRAF* | 15 | c.1799T>C | p.V600A |  |
| *BRAF* | 15 | c.1799T>G | p.V600G |  |
| *DDR2* | 18 | c.2304T>A | p.S768R |  |
| *MEK1* | 2 | c.167A>C | p.Q56P |  |
| *MEK1* | 2 | c.171G>T | p.K57N |  |
| *MEK1* | 2 | c.199G>A | p.D67N |  |
| *AKT* | 2 | c.49G>A | p.E17K |  |
| *ERBB2* | 19 | c.2264T>C | p.L755S |  |
| *ERBB2* | 19 | c.2305G>C | p.D769H |  |
| *ERBB2* | 20 | c.2324_2325ins12 | p.A775_G776insYVMA |  |
| *ERBB2* | 20 | c.2325_2326ins12 | p.A775_G776insYVMA |  |
| *ERBB2* | 20 | c.2326G>T | p.G776C | 21 |
| *ERBB2* | 20 | c.2326delG>TTAT | p.G776>LC | 21 |
| *ERBB2* | 20 | c.2326delG>TTGT | p.G776>LC | 21 |
| *ERBB2* | 20 | c.2326G>A | p.G776S |  |
| *ERBB2* | 20 | c.2326G>CTTT | p.G776>LC |  |
| *ERBB2* | 20 | c.2326_2327insTGT | p.G776>VC | 22 |
| *ERBB2* | 20 | c.2326_2327insTGT | p.G776>VC | 22 |
| *ERBB2* | 20 | c.2326_2327insTTT | p.G776>VC | 22 |
| *ERBB2* | 20 | c.2326_2327insTCT | p.G776>VC | 22 |
| *ERBB2* | 20 | c.2331_2332insTGTGGG | p.V777_G778insCG |  |
| *ERBB2* | 20 | c.2339_2340insTGGCTCCCC | p.P780_Y781insGSP |  |
| *ERBB2* | 20 | c.2339_2340insGGGCTCCCC | p.P780_Y781insGSP |  |
| *ALK* | 23 | c.3586C>A | p.L1996M |  |
| *ALK* | 23 | c.3604G>A | p.G1202R |  |
| *ALK* | 23 | c.3617C>A | p.S1206Y |  |
| *ALK* | 25 | c.3806G>C | p.G1269A |  |
