# Supplementary material for: Molecular profiling of lung cancer specimens and liquid biopsies using MALDI-TOF mass spectrometry
Source: Diagn Pathol. 2018 Jan 12;13:4. doi: 10.1186/s13000-017-0683-7 (PMC6389067; doi:10.1186/s13000-017-0683-7)
Supplement: Supplementary file 3 — Molecular profile of NSCLC mutated cases. For each detected mutation, the number of positive cases and the diagnosis are reported. (DOCX 14 kb) [file 13000_2017_683_MOESM3_ESM.docx]

Supplementary Table 3. Molecular profile of NSCLC mutated cases. For each detected mutation, the number of positive cases and the diagnosis are reported.

| Tumor diagnosis | Gene | N°. of mutated tumors | Mutation | N° of cases with each mutation |
| --- | --- | --- | --- | --- |
| ADC | *EGFR* | 12 | L858R | 3 |
|  |  |  | L858R+T790M | 2 |
|  |  |  | L747_T751delLREAT | 1 |
|  |  |  | E746_A750delELREA | 3 |
|  |  |  | E746 ^a^ | 1 |
|  |  |  | L747 ^b^ | 1 |
|  |  |  | S768I | 1 |
|  | *KRAS* | 29 | G12C ^c^ | 14 |
|  |  |  | G12V | 5 |
|  |  |  | G12D ^d^ | 4 |
|  |  |  | G12F | 2 |
|  |  |  | G13D | 2 |
|  |  |  | Q61H | 2 |
|  | *ERBB2* | 3 | A775_G776insYVMA | 1 |
|  |  |  | G776>LC ^c^ | 2 |
|  | *AKT* | 2 | E17K ^d^ | 2 |
|  | *BRAF* | 1 | V600E | 1 |
| SCC | *PIK3CA* | 3 | H1047R | 2 |
|  |  |  | E542K | 1 |
| NSC | *KRAS* | 1 | G12A | 1 |

^a^ The mutation detected was not distinguishable among K745_E746insIPVAIK,

K745_E746insTPVAIK, E746_E749delELRE, E746_T750>IP.

^b^ The mutation detected was not distinguishable among L747P, L747_A750>P, L747_T751>PT,

L747_S752del, L747_S752>Q, L747_P753>Q.

^c,d^ mutations concomitant in one case.
