# Supplementary figures and images for: Molecular profiling of lung cancer specimens and liquid biopsies using MALDI-TOF mass spectrometry
Source: Diagn Pathol. 2018 Jan 12;13:4. doi: 10.1186/s13000-017-0683-7 (PMC6389067; doi:10.1186/s13000-017-0683-7)

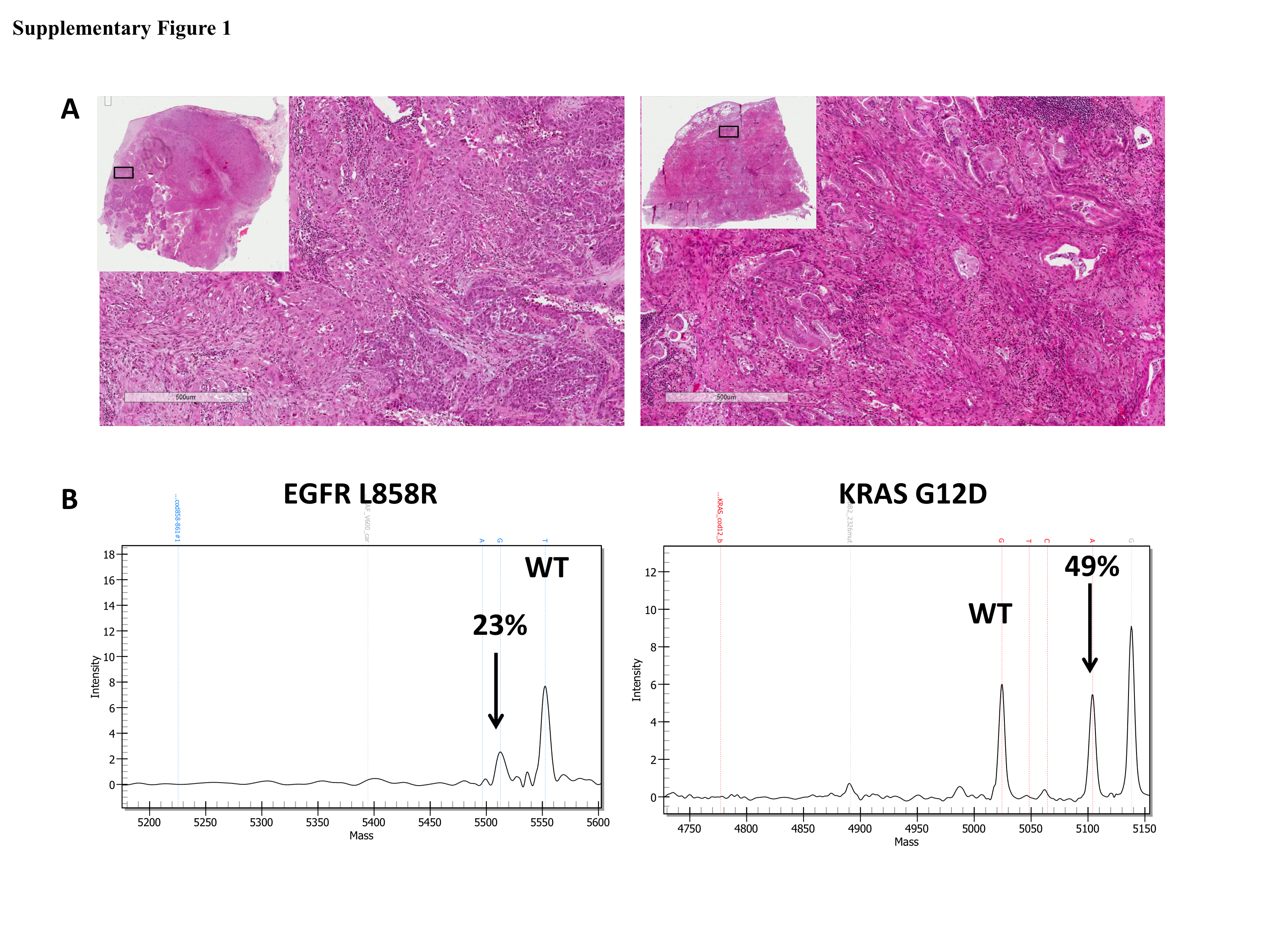

Supplement: Supplementary file 4 — H&E staining of two ADCs cases and the corresponding mutation MS spectra. A H&E staining of two ADCs cases. 5X zoom of the rectangular area is shown. The percentage of cancer cells is higher than 70% in both samples. B MS spectra of the two ADCs cases harboring EGFR L858R and KRAS G12D mutations, respectively. The mutated alleles are pointed out by black arrows and the corresponding percentages are reported in each spectrum. (TIFF 26330 kb) [file 13000_2017_683_MOESM4_ESM.tiff]
